# Advances in Photoluminescence and Quenching Mechanism of Carbon Dots

**DOI:** 10.3390/nano16110686

**Published:** 2026-06-01

**Authors:** Qingyun Xiong, Hafiz M. Ahsen Ilyas, Weiyu Cao, Jinping Xiong

**Affiliations:** 1 College of Materials Science and Engineering, Beijing University of Chemical Technology, Beijing 100029, China; xiongqy@mail.buct.edu.cn (Q.X.); hafizahsen2019@outlook.com (H.M.A.I.); 2 Beijing Research Institute of Uranium Geology, Beijing 100029, China

**Keywords:** carbon dots, photoluminescence mechanism, fluorescence quenching, quantum confinement, surface state, molecular fluorophore, crosslink-enhanced emission

## Abstract

Carbon dots (CDs) are zero-dimensional carbon nanomaterials with sizes below 10 nm, with high fluorescence quantum yields, variable emission colours, and excellent photostability. Due to their different structural origins and complex surface chemicals, CDs display complex photoluminescence behaviors (PL) and different fluorescence suppression responses. This review systematically summarizes recent advances in understanding the PL mechanisms of CDs, including carbon-core emission, surface emission, molecular emission and crosslink emission. In addition, fluorescence quenching processes triggered by various analytical techniques are discussed, including dynamic quenching, static quenching, Förster resonance energy transfer (FRET), photoinduced electron transfer (PET), and the inner filter effect (IFE). Emphasis is placed on mechanistic understanding and experimental differentiation strategies. A clear understanding of these fundamental mechanisms is essential for optimizing the fluorescence properties of CDs and the design of highly sensitive and selective fluorescence sensors. Finally, potential research directions and applications of CDs based on these mechanical insights are also highlighted.

## 1. Introduction

Carbon dots (CDs), a recently recognized member of the family of carbon nanomaterials, are quasi-spherical nanoparticles with a diameter of typically less than 10 nm. It consists mainly of hybridized sp^2^/sp^3^ carbon atoms and is decorated with rich surface functional groups such as hydroxyl, carboxyl and amino groups. Since its accidental discovery during the purification of single-wall carbon nanotubes in 2004 [1], CDs have attracted considerable interest in applications in sensing, bioimaging, optical electronics, and catalysis [2,3,4,5,6,7,8]. Compared to conventional semiconductor quantum dots (e.g., CdTe, PbS) and organic fluorescent dyes, CDs offer several advantages, including low toxicity, high biocompatibility, excellent photosafety and simple synthesis of various precursors [9,10,11].

A distinguishing characteristic of CDs is their diverse emission behaviors, which stem from their complex internal structures and surface states [2]. Previous studies [3,11] have suggested that the photoluminescence of CDs is often governed by multiple mechanisms, including the quantum confinement effect, surface-state emission, molecular fluorophore emission, and crosslink-enhanced emission. Meanwhile, the fluorescence of CDs can be efficiently quenched by various analytes (e.g., metal ions, small organic molecules, biomacromolecules) through different pathways, such as dynamic quenching, static quenching, FRET, PET, and IFE. These quenching mechanisms serve as the fundamental basis for the development of CD-based analytical sensors.

Notably, CDs exhibit rich fluorescent centers due to the wide range of precursor sources and complex structural compositions. Different luminescent centers correspond to distinct photoluminescence mechanisms: the quantum confinement effect analogous to semiconductor quantum dots, surface-state emissions associated with surface defects and dopant atoms, as well as molecular-state and crosslink-enhanced emissions corresponding to molecular fluorophores and sub-fluorophores, respectively [12]. Therefore, a comprehensive understanding of the photoluminescence mechanism is a prerequisite for improving the fluorescence properties (e.g., quantum yield, emission tunability) of CDs. Simultaneously, elucidating the fluorescence quenching mechanism is essential for designing and optimizing CD-based sensors with enhanced performance.

Unlike previous reviews that primarily summarize reported mechanisms, this work emphasizes mechanism discrimination and critical evaluation, providing practical guidance for distinguishing photoluminescence and quenching pathways using experimental criteria. Additionally, this review addresses ongoing controversies in the field, particularly regarding the role of quantum confinement, and highlights the limitations of existing interpretations.

This review highlights current progress in understanding the photoluminescence and fluorescence quenching mechanisms of carbon dots (CDs). The discussion begins with an overview of the PL mechanisms, categorized according to the luminescent centers of CDs, including carbon-core-state emission, surface-state emission, molecular-state emission (with emphasis on solvent effects), and crosslink-enhanced emission (CEE). Following this, the major fluorescence quenching pathways are systematically summarized, supported by representative examples and experimental characterization techniques. Finally, current challenges and upcoming research prospects in this field are outlined, providing a comprehensive reference for researchers working on CD-based photoluminescent materials and sensing applications.

## 2. Photoluminescence Mechanisms of Carbon Dots

It is important to note that CDs are inherently heterogeneous systems containing multiple emissive species, including carbon cores, surface states, molecular fluorophores, and oligomeric structures. This heterogeneity leads to batch-to-batch variability and complicates mechanistic interpretation. Purification techniques such as dialysis, chromatography, and electrophoresis are therefore critical for isolating distinct emissive components. Moreover, most reported optical measurements are ensemble-averaged, which may obscure contributions from individual emitters. Advanced techniques such as single-particle spectroscopy and high-resolution separation methods are essential for resolving the “multiple-emitter” nature of CDs and improving the reliability of mechanistic assignments in the Photoluminescence Mechanism of CDs [13,14].

CDs exhibit diverse types and structural features, leading to multiple luminescent origins. The structural units that can act as luminescent centers in CDs include incompletely conjugated and size-limited π-structural domains, surface defects and doping sites, molecular fluorophores, and sub-fluorophores [12]. Thus, a single photoluminescence mechanism (e.g., quantum confinement effect) cannot fully explain the luminescent behaviors of all CDs. In this part, the photoluminescence mechanisms of CDs are discussed in an integrated manner based on their luminescent centers.

### 2.1. Carbon-Core-State Emissions

Carbon-core-state emission is predominantly observed in carbon quantum dots (CQDs) and graphene quantum dots (GQDs), and its mechanism can be understood through the quantum confinement effect, similar to that in traditional semiconductor quantum dots. The carbon core of these CDs is composed of conjugated sp^2^ carbon domains. When the effective conjugation length [15] of these sp^2^ domains is reduced to the nanometer scale, the quasi-continuous electronic energy levels near the Fermi level become discretized. The luminescence of the carbon core is largely determined by the size of the conjugated sp^2^ domains: as the effective conjugation length increases, the energy gap between the highest occupied molecular orbital (HOMO) and the lowest unoccupied molecular orbital (LUMO) decreases, resulting in a redshift in the emission wavelength [16].

However, the applicability of this interpretation to CDs remains highly debated. Unlike classical semiconductor quantum dots (e.g., CdSe), graphitic carbon materials such as graphene are intrinsically semi-metallic, exhibiting zero bandgap in their ideal form. As a result, the existence of a well-defined quantum confinement effect in such systems is not straightforward. Although finite-sized graphene fragments (graphene quantum dots) may exhibit edge-induced or defect-induced bandgaps, these are strongly influenced by edge structure, functionalization, and disorder rather than purely by size [17,18,19].

Several studies have reported size-dependent photoluminescence behavior in CDs and attributed this to quantum confinement. For example, emission redshifts with increasing particle size have been observed in graphene quantum dots and carbon quantum dots synthesized via top–down or bottom–up approaches. While such trends are qualitatively consistent with confinement-based models, size-dependent emission alone does not constitute definitive evidence of quantum confinement. In many cases, variations in surface chemistry, oxidation level, and defect density occur simultaneously with changes in particle size, making it difficult to isolate the effect of core size [20,21,22,23].

A growing body of evidence suggests that surface states and defect-related emissive centers play a dominant role in determining the photoluminescence of CDs. As particle size changes, the relative contribution of these surface states may also evolve, leading to apparent size-dependent emission shifts. For instance, smaller particles often possess higher surface-to-volume ratios and more abundant surface functional groups, which can introduce higher-energy emissive states and produce a blueshift in emission. Conversely, larger particles may favor more conjugated domains and lower-energy emissive states, resulting in redshifted emission. These effects can mimic the optical signatures traditionally attributed to quantum confinement [24,25].

This issue is not unique to carbon-based systems. Similar misinterpretations have been reported in other nanomaterials, such as silicon carbide quantum dots, where emission behavior initially attributed to quantum confinement was later reassigned to defect- and surface-state emission. These findings highlight the importance of rigorous mechanistic validation rather than relying solely on phenomenological observations such as emission shifts [26,27].

Therefore, while the concept of carbon-core-state emission remains useful for describing certain classes of CDs particularly those with relatively well-developed sp^2^ domains—it should be applied with caution. A definitive assignment of quantum confinement requires quantitative correlation between size and bandgap, supported by complementary evidence such as electronic structure calculations, controlled synthesis, and spectroscopic validation. In many reported systems, the photoluminescence of CDs is more accurately described as arising from a complex interplay between core electronic structure, surface states, and molecular emissive species, rather than a purely confinement-driven mechanism [28].

Although a redshift in emission with increasing particle size is observed, this trend alone does not constitute definitive evidence of quantum confinement, as similar behavior may arise from variations in surface states and defect distributions [29].

**Figure 1:**
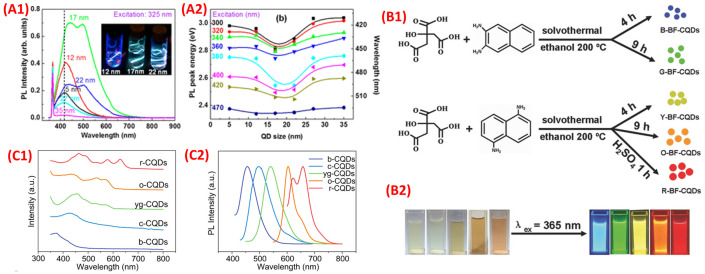
(**A1**) Photoluminescence (PL) spectra of graphene quantum dots (GQDs) with average particle sizes between 5 and 35 nm measured under 325 nm excitation in deionized water. The inset presents fluorescence colors of three typical GQD samples (12, 17, and 22 nm), demonstrating the size-dependent emission behavior. (**A2**) Dependence of PL peak position on excitation wavelength (300–470 nm) for GQDs with an average size of approximately 5–35 nm [30]. (**B1**) Schematic illustration of the synthesis of multicolor band fluorescence carbon quantum dots (MCBF-CQDs), showing tunable emission from blue to red via solvothermal reaction of citric acid (CA) and diaminonaphthalene (DAN). (**B2**) Optical photographs of MCBF-CQDs under visible light (left) and fluorescence images captured under 365 nm UV irradiation (right) [31]. (**C1**) UV–visible absorption spectra of CQDs; (**C2**) Corresponding normalized photoluminescence spectra of CQDs [32].

Numerous experimental studies have confirmed the size-dependent luminescence of carbon-core-state CDs. Kim et al. [30] prepared GQDs of different sizes by cutting graphene sheets and originate (indicate that the best (or strongest) emission wavelength positions) that the optimal emission peak positions of GQDs varied with their sizes under the same excitement. The fluorescence emission of GQDs at 325 nm (Figure 1A) was found to redshift with increasing graphene domain size. This occurs because larger graphene domains enhance electron delocalization and create a more uniform energy distribution, resulting in longer-wavelength emission. Yuan et al. [31] synthesized multicolor CQDs by varying the solvent type and reaction time during the solvothermal treatment of citric acid (CA) and diaminonaphthalene (DAN). The emission peaks of the resulting blue (B-), green (G-), yellow (Y-), orange (O-), and red (R-) CQDs were observed at 430, 513, 535, 565, and 604 nm, respectively. TEM analysis revealed average particle sizes of 1.95, 2.41, 3.78, 4.90, and 6.68 nm, respectively, clearly demonstrating the size-dependent luminescence associated with the carbon-core states of CDs (Figure 1B). Similarly, Wang et al. [32] prepared multicolor CQDs using o-phenylenediamine (o-PDA) under acid reagent engineering. The introduction of acidic reagents increased surface electron-withdrawing groups and promoted particle growth, resulting in a redshift of emission (Figure 1C). The optical band gap, calculated using Egopt = 1240/λ, gradually decreased from 2.76 eV to 1.88 eV with increasing CQD size, further confirming the role of quantum confinement in carbon-core-state emissions.

It is important to note that the attribution of photoluminescence in carbon dots to quantum confinement remains controversial. Unlike conventional semiconductor quantum dots, graphitic carbon structures such as graphene are semi-metallic and do not possess an intrinsic bandgap. While size-dependent emission has often been interpreted as evidence of quantum confinement, such behavior alone is not sufficient to confirm this mechanism. In many cases, emission shifts may instead arise from changes in surface defect states, functional groups, or localized emissive centers that vary with particle size and synthesis conditions. Similar misinterpretations have been reported in other nanomaterial systems, such as silicon carbide quantum dots, where emission was initially attributed to quantum confinement but later reassigned to defect-related states. Therefore, caution should be exercised when invoking quantum confinement as the dominant mechanism in CDs, and alternative explanations based on surface and molecular emissive states should be carefully considered [33,34].

### 2.2. Surface-State Emissions

The surface-state emission mechanism is demonstrated by the hybridization state of the art carbon backbone and the attached chemical groups, which explains the changes in luminescent sites caused by variations in electronic energy levels. Surface states have a more significant impact on GQDs than on CQDs. Factors influencing surface-state emission include surface configurations (e.g., functional groups with different oxidation levels, surface defects, edge states) and dopant atoms.

#### 2.2.1. Surface Configuration

**Figure 2:**
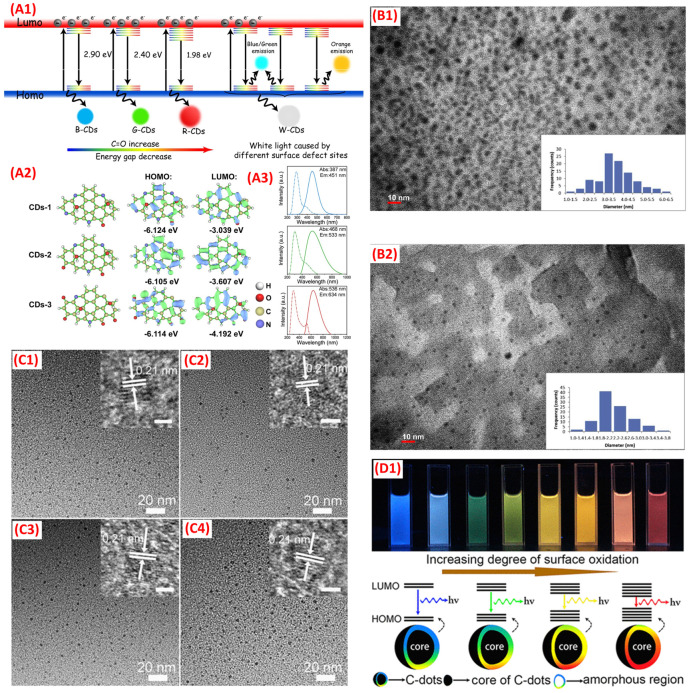
(**A1**) Schematic representation of tricolor and white-light emission behavior from CDs; (**A2**) Time-dependent density functional theory (TD-DFT) calculations showing the structural model of CDs composed of nine fused benzene rings, along with the corresponding HOMO and LUMO; (**A3**) Photoluminescence (PL) and UV–Vis absorption spectra of CDs-1, CDs-2, and CDs-3 [35]. TEM images of fractions (**B1**,**B2**), with scale bars indicating 10 nm [36]. Overview TEM images of (**C1**) Sample 1, (**C2**) Sample 2, (**C3**) Sample 3, and (**C4**) Sample 4, demonstrating uniform dispersion and narrow size distribution. Insets show the corresponding high-resolution TEM (HRTEM) images, revealing clear crystalline lattices with a d-spacing of 0.21 nm, characteristic of the graphitic carbon core structure. (Scale bars: main images 20 nm; insert inset scale bar size, 0.21 nm [25]. (**D1**) Conceptual model illustrating tunable photoluminescence of CDs as a function of different oxidation levels [25].

Surface configuration, including the nature and content of the surface functional groups, surface defects, and edge states, directly modulates the electronic energy levels of CDs, thereby affecting their luminescent properties. Li et al. [35] constructed three 9-membered benzene ring models for CDs with different nitrogen and oxidation substitution degrees: low-oxidized high-nitrogen-substituted (CDs-1), medium-oxidized medium-nitrogen-substituted (CDs-2), low-nitrogen and high-oxidized substituted (CDs-3). The calculated (LUMO) energy levels were −3.039 eV, −3.607 eV, and −4.192 eV, respectively (Figure 2A). The change in the conformation of the CD basal plane and the oxygen-containing functional groups attached to the edge sites transformed the CD structure from planar to bowed, reducing the geometric stiffness and leading to a wider range of electronic rearrangements. This geometric change was identified as a key factor resulting in the small spatial detour overlap between (HOMO) and (LUMO) [25,36,37,38]. Ding et al. [25] isolated a series of CDs with similar sizes but different luminescent colors (Figure 2C). While emission, the color shifting from blue to red, the hydroxyl content on the CD surface gradually increased, indicating that the multicolor luminescence of these CDs originated from surface-state emission. The energy gap of the surface states decreased with the enhancement of surface oxidation (Figure 2D). In another study, Zhou et al. [36] separated the crude microwave-assisted reaction products of CA and o-PDA using size-exclusion chromatography, obtaining three CD fractions with different sizes. Further characterization revealed that the fluorescence differences among these CDs were closely related to their surface states (determined by surface functional groups) rather than their particle sizes (Figure 2B), highlighting the leading role of surface configuration in surface-state emission.

#### 2.2.2. Dopant Atoms

**Figure 3:**
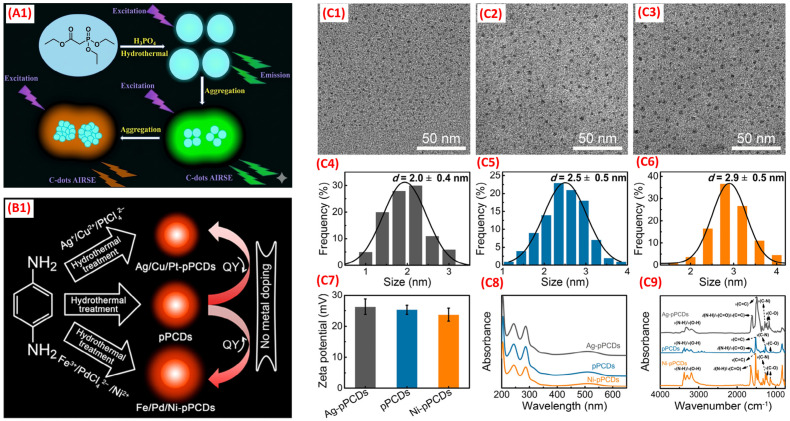
(**A1**) Schematic diagram illustrating the synthesis route of phosphorus-doped carbon dots (P-CDs) and the aggregation-induced room-state emission (AIRSE) phenomenon observed with increasing particle concentration [44]. (**B1**) Conceptual illustration showing that specific metal ions may assist or act as catalytic centers to promote element doping during the CD formation process [46]. Morphological and physicochemical characterization of CDs: (**C1**–**C3**) TEM images and (**C4**–**C6**) corresponding particle size distribution profiles of Ag–pPCDs, pPCDs, and Ni–pPCDs; (**C7**) Zeta potential distribution histogram; (**C8**) UV–Vis absorption spectra; and (**C9**) FTIR spectra of the three CD samples [46].

Doping-induced surface defects provide an effective method to tune the emission wavelengths of CDs by introducing additional energy levels between the HOMO and LUMO. Nitrogen is the most widely used non-metal dopant, present in forms such as amino, pyridine, pyrrole, and graphitic nitrogen. Pyridine nitrogen typically contributes to a blue shift in fluorescence emission, whereas higher graphitic nitrogen content correlates with redshifted absorption and emission spectra of CDs [39,40,41]. Sulfur doping introduces new band gaps and enhances coordination [42], boron doping facilitates charge transfer processes [43], and fluorine doping supports fluorescence stability under varying pH conditions [44]. Additionally, Li et al. [45] reported AIRSE (Aggregation-induced red-shifted emission) in phosphorus-doped CDs (Figure 3A). Phosphorus doping promotes the formation of electron-rich regions within CDs. As CD concentration and particle size increase, and the electronic system expands, a π–π conjugation-like effect emerges, leading to redshifted emission.

Metal ion incorporation during CD synthesis can also influence luminescent properties. Hua et al. [46] investigated the hydrothermal synthesis of o-PDA-derived CDs with various metal ions as dopants. Although the final products contained no detectable metal, the existence of different metal ions significantly affected the quantum yields of the CDs (Figure 3B,C). This suggests that metal ions may act as catalysts during CD formation, indirectly modulating surface states and the resulting luminescent behavior of CDs.

### 2.3. Molecular-State Emissions and Solvent Effects

#### 2.3.1. Molecular-State Emissions

Molecular-state emission in carbon dots (CDs) originates from discrete molecular fluorophores or small organic species either attached to the surface or formed during synthesis. These emissive centers typically exhibit excitation-independent emission, narrow emission bands, and strong dependence on solvent polarity and chemical environment [47]. During the bottom–up synthesis process, small molecules or polymers undergo intermolecular/intramolecular dehydration and intramolecular cyclization at relatively low temperatures, forming small organic molecules with strong fluorescence (molecular fluorophores). These molecular fluorophores are bonded to the surface or embedded in the carbon core of CDs, serving as the main luminescent centers for molecular-state emission [48].

**Figure 4:**
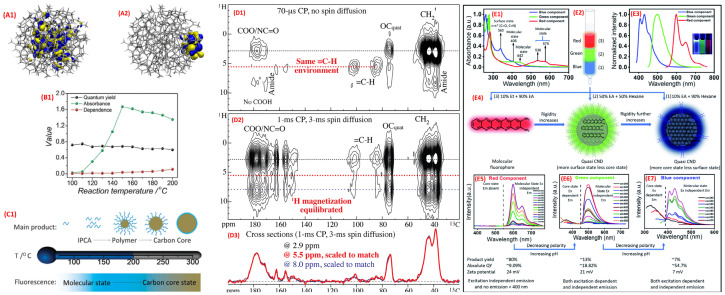
(**A1**) Typical molecular orbital distributions (isodensity (isodensity surface set refers to a constant electron density surface used to visualize molecular orbitals in computational simulations) surface set at 0.01 e^−^ Å^−3^) representing band-like electronic states; (**A2**) surface-associated electronic states of a ~2 nm carbon nanodot [49]. (**B1**) Absorption characteristics (Abs), fluorescence quantum yield (QY), and Dep parameter values of carbon dot solutions synthesized at various reaction temperatures [50]. (**C1**) Schematic representation illustrating the formation process and structural differences in CDs produced at different hydrothermal synthesis temperatures [50]. HETCOR ^1^H–^13^C NMR spectra of carbon dots derived from citric acid and ethylenediamine: (**D1**) spectrum obtained using a short cross-polarization (CP) contact time of 0.07 ms; (**D2**) spectrum collected using 3 ms ^1^H spin diffusion followed by 1 ms CP; (**D3**) corresponding cross-sectional spectra extracted from (**D2**) at chemical shifts of 2.9 ppm (black), 5.5 ppm (red), and 8.0 ppm (blue) [51]. (**E1**) Absorption spectra of three isolated fluorescent components; (**E2**) schematic diagram illustrating separation of red, green, and blue emitting fractions using column chromatography; (**E3**) emission spectra of the separated fractions (inset shows corresponding UV emission photographs); (**E4**) proposed structural models for red, green, and blue emissive components, where the red fraction is associated with molecular fluorophore structures, while the green and blue fractions exhibit quasi-carbon nanodot (CND) structures with a more developed core structure in the blue fraction; (**E5**–**E7**) emission spectra obtained by varying excitation wavelengths for red, green, and blue fractions, respectively. The red fraction shows excitation-independent emission, while green and blue fractions display both excitation-dependent and excitation-independent emission characteristics [52].

Margraf et al. [49] investigated the electronic structure of carbon nanodots using computational simulations. At lower synthesis temperatures, amide-containing molecular fluorophores were the main luminescent centers. While increasing the temperature, the organic fluorophores were gradually consumed and carbonized, and the luminescence of CDs were dominated to the carbon-core-state emission (Figure 4A). Sang et al. [50] investigated the temperature dependence of absorption peak quantum yield (QY), intensity (Abs), and excitation wavelength dependence (Dep) of hydrothermal products of CA and ethylenediamine (EDA) (Figure 4B). The results indicated that the products contained molecules, polymer clusters, and carbon cores at different temperatures. At 140 °C, the molecular fluorophore was identified as imidazolidine-2,4,5-trione (IPCA) (Figure 4C). Subsequently, Duan et al. [51] indicate the presence of IPCA and its derivatives in the microwave-assisted reaction products of CA and EDA using solid-state nuclear magnetic resonance (NMR) techniques. Quantitative ^13^C NMR analysis showed that the content of IPCA in CDs reached up to 18 ± 2 wt% (Figure 4D). Soni et al. [52] isolated a red-emissive molecular fluorophore (QXPDA) from o-PDA-derived CPDs, which accounted for 80% of the CPD mass with an absolute quantum yield of 9%. The emission spectrum of QXPDA was completely independent of excitation wavelength (Figure 4E), further confirming the existence of molecular-state emission in CDs.

#### 2.3.2. Solvent Effects

Fluorescence originating from molecular states is generally more sensitive to the solvent environment than emissions from carbon-core or surface states. Solvent-induced shifts in the fluorescence spectrum can arise from two main mechanisms: general solvation effects and specific solvation effects [53,54,55,56,57]. General solvation effects are primarily governed by changes in solvent polarity, whereas specific solvation effects involve hydrogen bonding, internal charge transfer, excited-state proton transfer, and conformational rearrangements.

**Figure 5:**
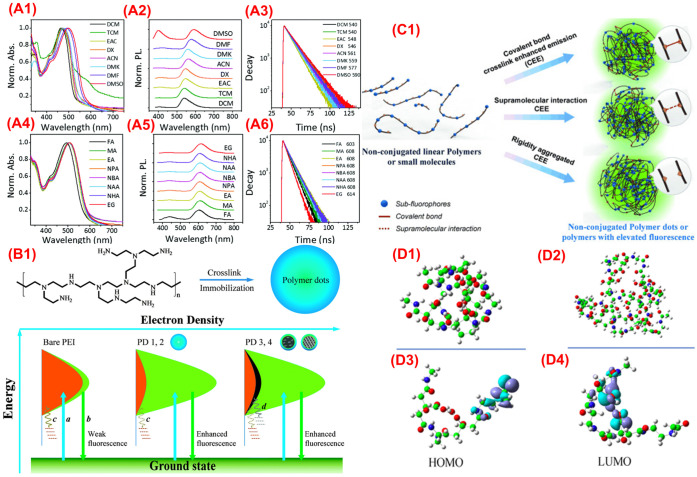
Normalized UV–Vis absorption spectra, photoluminescence emission spectra, and fluorescence lifetime decay profiles of CDs recorded in aprotic solvents (**A1**–**A3**) and protic solvents (**A4**–**A6**) respectively [58]. (**B1**) Conceptual schematic showing crosslink-enhanced emission (CEE) originating from covalent crosslinking, supramolecular interactions, and/or rigidity-enhanced aggregation effects in non-conjugated polymer dots or polymer materials. Sub-fluorophore groups may be distributed along the polymer backbone or attached to the side chains of linear non-conjugated polymers. The interaction sites generated through covalent or supramolecular bonding may also serve as emission-active sub-fluorophore centers [61]. (**C1**) Schematic diagram illustrating the CEE-related photoluminescence mechanism for pristine PEI and PDs 1–4. Electrons excited from the ground state are trapped by amino-related electronic states, subsequently returning to the ground state via radiative recombination or undergoing vibration- and rotation-mediated non-radiative relaxation [62]. Energy-optimized molecular structures of (**D1**) two dimer chains (*n* = 2) and (**D2**) one decamer chain (*n* = 10). (**D3**) Spatial distribution of the highest occupied molecular orbital (HOMO) and (**D4**) lowest unoccupied molecular orbital (LUMO) contributing to fluorescence emission properties [63].

Zhang et al. [58] reported that CDs with molecular-state emission displayed markedly different fluorescence behaviors in protic versus aprotic solvents (Figure 5A). In aprotic solvents, increasing solvent polarity caused a gradual redshift of the emission peak from 540 nm to 590 nm. In contrast, in protic solvents, the emission peak remained between 608 and 615 nm, showing minimal sensitivity to polarity. This suggests that hydrogen bonding in protic solvents mitigates the influence of solvent polarity on emission. In aprotic solvents, dipole–dipole interactions modulate electron density distributions; larger dipole moments enhance charge transfer, reduce the energy gap, and consequently induce redshifted absorption and emission spectra [59]. Hydrogen bonding between molecular fluorophores and protic solvents restricts collisions and conformational flexibility of the CDs, thereby suppressing solvent-induced spectral shifts [60].

### 2.4. Crosslink-Enhanced Emissions (CEE)

Molecular structures that contain only heteroatom-based double bonds (such as C=O, C=N, and N=O) or simple single-bond functional groups (including C–O and –NH_2_) generally show weak or negligible fluorescence. Interestingly, recent investigations have demonstrated that materials containing these nonconventional fluorophoric units can produce strong fluorescence after undergoing specific chemical or physical treatments [61,62,63,64,65]. To rationalize this unusual behavior, Yang and co-workers introduced the concept of crosslink-enhanced emission (CEE) [61].

The CEE effect supports the photoluminescence performance of carbonized polymer dots (CPDs) by restricting polymer chain motion and introducing additional electronic energy states [61]. When polymer chains are immobilized through covalent crosslinking or intermolecular aggregation, the vibrational and rotational motions of sub-fluorophore units are significantly suppressed. This restriction reduces non-radiative energy dissipation and favors radiative recombination pathways [66,67]. Furthermore, crosslinking or aggregation changes the spatial arrangement of functional groups, enabling orbital overlap and electronic coupling between neighboring groups. This interaction leads to energy level redistribution, allowing electrons to undergo efficient radiative transitions [68]. According to the dominant mechanism, CEE can generally be classified into immobilization-induced CEE and confined-domain CEE.

#### 2.4.1. Immobilization CEE

Covalent bonding, supramolecular interactions, and rigid aggregation are effective immobilization modes for CEE (Figure 5B) [61]. Covalent bonding corresponds to strong and stable intramolecular interactions, and covalent crosslinking results in a more rigid structure. Zhu et al. [62] designed four polyethyleneimine (PEI)-based crosslinked CD systems: PEI crosslinked with carbon tetrachloride (CTC) as the crosslinker (PDs-1), hydrothermal PEI products (PDs-2), PEI immobilized on lattice-free CD surfaces (PDs-3), and PEI immobilized on CQD surfaces (PDs-4) (Figure 5C). PDs-1 exhibited the optimal fluorescence properties because PEI contains potential fluorophores (secondary and tertiary amines). Covalent crosslinking inhibits the vibration and rotation of sub-fluorophores, increasing radiative transitions. Temperature-dependent fluorescence studies confirmed this mechanism: the fluorescence intensity of CPDs reduced with cumulative temperature, as elevated temperatures enhance the shaking and turning of amine groups, promoting non-radiative decay.

Supramolecular interactions (including hydrogen bonding, van der Waals forces, π-π stacking, host–guest interactions, and coordination) can also induce CEE. In the low-temperature synthesis of bright blue CDs from CA and EDA [63], density functional theory (DFT) calculations revealed that light-induced charge transmission between amide and carboxyl groups was the origin of blue fluorescence. Hydrogen bonding-mediated supramolecular interactions between polyamide chains formed a rigid network structure, further facilitating radiative processes (Figure 5D).

#### 2.4.2. Domain-Limited CEE

Crosslinking and entanglement confine luminophores to restricted domains, resulting in proximity or even overlay of electron clouds. Fixed-domain orbitals or orbital groups interact directly through space or tortuously through the chemical bonds, modulating the luminescent properties of CDs.

**Figure 6:**
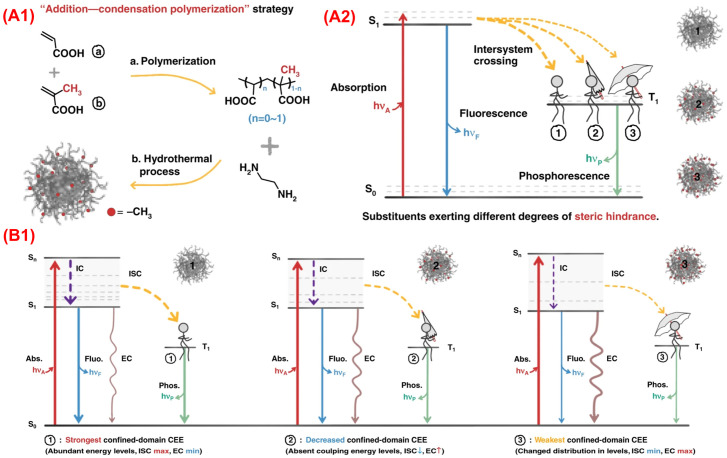
Overall schematic model for exploring confined-domain crosslink-enhanced emission (CEE) behavior in carbonized polymer dots (CPDs). (**A1**) Illustration of the “addition–condensation polymerization” synthetic route; (**A2**) Jablonski-type diagram describing potential photoluminescence (PL) pathways of CPDs containing substituents with different steric hindrance levels (1: negligible steric hindrance; 2: moderate steric hindrance; 3: significant steric hindrance) [69]. (**B1**) Schematic diagram illustrating how confined-domain CEE influences the electronic energy level structure of CPDs (Phos.: phosphorescence; IC: internal conversion; Fluo.: fluorescence; Abs.: absorption) [69].

Tao et al. [69] investigated the effect of spatial interactions on the fluorescence of CPDs in confined domains using an “addition-condensation polymerization” strategy (Figure 6A). CPDs were prepared by copolymerizing adipic acid (AA) and methyl adipate (AACH_3_) in different ratios, followed by hydrothermal treatment with EDA. Three CPD samples with AA/AACH_3_ ratios of 1/0, 1/1, and 0/1 (denoted as CPDsCH_3_-1, CPDsCH_3_-2, and CPDsCH_3_-3) were selected for detailed studies. The results showed that methyl groups regulated the configuration of luminescent units. With increasing methyl content, the degree of planar distortion increased, leading to changes in the energy level structure [70,71]. The radiative transition modes of CPDsCH_3_-3 differed from those of CPDsCH_3_-1 and CPDsCH_3_-2 (Figure 6B). Domain-limited CEE altered the photoluminescence properties of CPDsCH_3_-1 and CPDsCH_3_-2, ultimately inducing energy level changes in CPDsCH_3_-3. The strongest domain-limited CEE was observed in CPDsCH_3_-1 (no steric hindrance), characterized by abundant energy levels, maximum intersystem crossing (ISC), and minimum internal conversion (IC). In contrast, CPDsCH_3_-3 (large steric hindrance) exhibited the weakest domain-limited CEE, with changed energy level distribution, minimum ISC, and maximum IC.

### 2.5. TD-DFT & PL

Time-dependent density functional theory (TD-DFT) has emerged as an important theoretical tool for understanding the photoluminescence mechanisms of carbon dots. TD-DFT calculations enable the analysis of excited-state properties, electronic transitions, and energy level distributions, providing insight into the origin of emission from different functional groups and core structures. In particular, TD-DFT has been widely used to distinguish between core-state and surface-state emission and to evaluate the contribution of molecular fluorophores. These theoretical studies complement experimental observations and are essential for validating proposed emission mechanisms [33].

**Figure 7:**
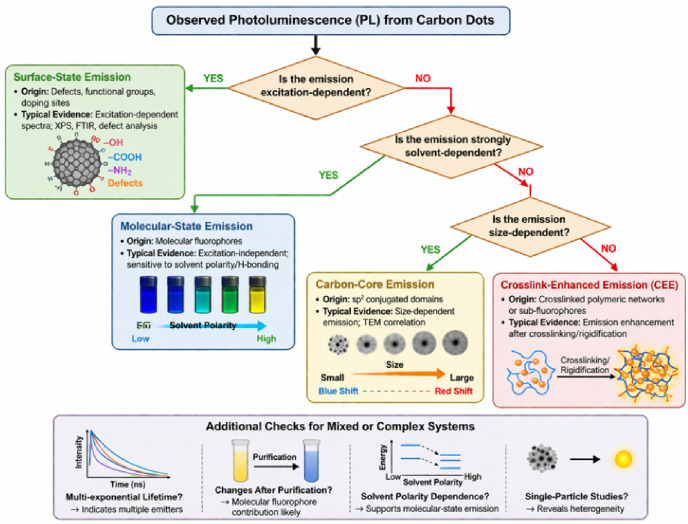
Decision-tree identifying PL mechanisms based on experimental signatures.

Although PL mechanisms are categorized into carbon-core, surface-state, molecular-state, and crosslink-enhanced emission, these mechanisms often coexist within a single CD system (Table 1) [72]. Their identification relies on combined spectroscopic signatures. For example, excitation-dependent emission is often associated with surface states, while excitation-independent emission suggests molecular fluorophores. Solvent sensitivity and polarity dependence typically indicate molecular-state contributions. Time-resolved fluorescence often reveals multiple decay components corresponding to different emissive centers. Additionally, synthesis routes (top–down vs. bottom–up), purification processes, and post-treatment significantly influence the dominant emission mechanism. Therefore, mechanistic assignment should be made cautiously based on multiple complementary techniques rather than a single observation. Furthermore, a decision-tree can be seen in Figure 7 to guide readers in identifying PL mechanisms based on experimental signatures [73,74].

Due to the structural diversity and heterogeneity of carbon dots, it is challenging to establish a universal photoluminescence mechanism. In many cases, multiple emissive centers coexist, and the observed optical properties represent a convolution of different contributions. Therefore, mechanistic interpretations should be made cautiously and supported by multiple complementary characterization techniques.

## 3. Quenching Mechanism of CDs

Tunable photoluminescence and excellent fluorescence stability are key characteristics that distinguish CDs from other fluorescent materials [75]. Understanding the fluorescence quenching mechanisms of CDs induced by different analytes and the corresponding changes in fluorescence properties is crucial for the development of high-performance sensors [76]. To date, five main quenching mechanisms have been identified: dynamic quenching, static quenching, Förster resonance energy transfer (FRET), photoinduced electron transfer (PET), and inner filter effect (IFE) [72].

### 3.1. Dynamic Quenching (DQ) and Static Quenching (SQ)

#### 3.1.1. Dynamic Quenching

Dynamic quenching occurs when CDs absorb photons and are promoted to the excited state, where they interact with quenching agents through collisions. The excited CDs then return to the ground state via energy transfer or electron transfer processes, accompanied by energy loss. This type of quenching primarily affects the excited-state population, leading to changes in fluorescence lifetime without significantly altering the absorption spectrum.

**Figure 8:**
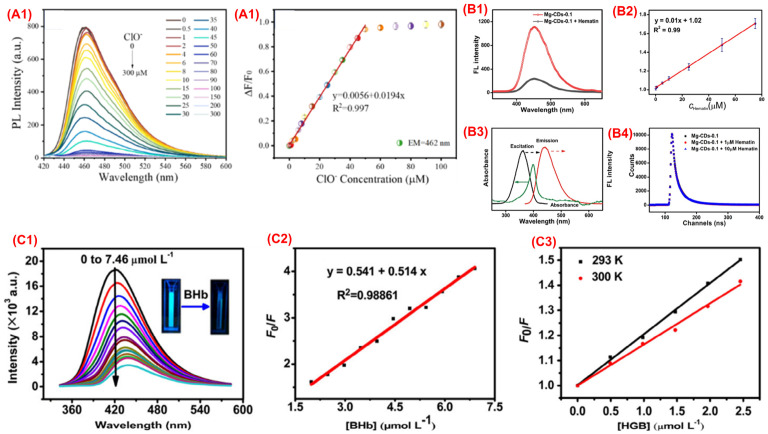
(**A1**) Fluorescence emission spectra of B-CDs were recorded with gradually increasing ClO^−^ concentrations in the range of 0–300 μM, showing concentration-dependent fluorescence quenching behavior. (**A2**) The relative fluorescence response (ΔF/F_0_) shows a linear relationship with ClO^−^ concentration over the range of 0.5–50 μM [77]. (**B1**) Fluorescence intensity of Mg-CDs-0.1 was compared in the absence and presence of hematin (500 μM). (**B2**) The detection sensitivity of hematin using Mg-CDs-0.1 was evaluated at pH 7.4 with an excitation wavelength of 314 nm. (**B3**) UV–Vis absorption spectrum of hematin was compared with the excitation and emission spectra of Mg-CDs-0.1. (**B4**) Fluorescence lifetime decay behavior of Mg-CDs-0.1 was analyzed before and after the addition of hematin [78]. (**C1**) Fluorescence emission spectra of nitrogen and sulfur co-doped carbon dots (N, S-CDs) were measured after adding different concentrations of bovine hemoglobin (BHb) (λex = 311 nm). The inset shows photographic images of N, S-CDs without and with 5.12 μmol L^−1^ BHb under UV light (360 nm). (**C2**) The corresponding Stern–Volmer plot of N, S-CDs in the presence of different concentrations of BHb, including temperature-dependent quenching behavior. (**C3**) The concentration of N, S-CDs used during the measurement was 5.0 μg mL^−1^ [79].

The Stern–Volmer equation, F_0_/F = K_SV_ [Q], is commonly applied to evaluate quenching efficiency [77,78,79,80], where K_SV_ is the Stern–Volmer constant, and [Q] is the quencher concentration, while Fand F_0_ characterize the fluorescence intensities before and after the addition of the quencher. In dynamic quenching, K_SV_ can be expressed as kq × τ_0_, in the absence of a quencher, where kq is the bimolecular quenching rate constant and τ_0_ is the fluorescence lifetime of CDs. Typically, kq for dynamic quenching is below 1.0 × 10^10^ M^−1^ s^−1^. Long et al. [77] studied the influence of hypochlorite (ClO^−^) on the fluorescence behavior of electrochemically synthesized CDs and found that the quenching did not alter the fluorescence lifetime, consistent with dynamic quenching. A linear relationship, ΔF/F_0_ = 0.0056 + 0.0194 C (ClO^−^) (R^2^ = 0.997), was observed for ClO^−^ concentrations in the range of 0.5–50 μM (Figure 8A), demonstrating the potential sensing applications of these CDs for ClO^−^ detection.

#### 3.1.2. Static Quenching

Static quenching takes place when quenching molecules interact with CDs to form non-emissive complexes in the ground state. Since this process alters the ground-state electronic structure, the UV–Vis absorption spectra of CDs usually change after quenching, whereas the fluorescence lifetime shows minimal variation. Yu et al. [78] examined the interaction between hematin and magnesium-doped CDs (Mg-CDs). Their results showed that the fluorescence intensity of Mg-CDs decreased proportionally with increasing hematin concentration in the range of 0.1–75 μM. Based on the Stern–Volmer analysis, the quenching constant (KSV) was calculated to be 3 × 10^4^ M^−1^. The measured fluorescence lifetime of Mg-CDs was 5.17 ns, corresponding to a calculated bimolecular quenching rate constant (kq) of 5.8 × 10^12^ M^−1^ s^−1^. This value is significantly higher than the typical upper limit associated with dynamic quenching, confirming that the dominant mechanism is static quenching (Figure 8B).

For static quenching, the K_SV_ value corresponds to the ground-state binding constant between the CDs and the quencher [80]. Temperature dependence is an important criterion for distinguishing static from dynamic quenching [72]. Higher temperatures destabilize ground-state complexes, reducing static quenching efficiency [81,82,83,84,85], whereas they increase the diffusion rate of quenchers, enhancing dynamic quenching. Wang et al. [79] reported that nitrogen and sulfur co-doped CDs (N, S-CDs) form ground-state complexes with bovine hemoglobin (BHb) via hydrophobic interactions. In the BHb concentration range of 1.49–6.96 μmol·L^−1^, the K_SV_ was 5.18 × 10^5^ M^−1^ and kq = 6.47 × 10^13^ M^−1^ s^−1^ (R^2^ = 0.9886). Upon increasing temperature, both F0/F and K_SV_ decreased, with equilibrium binding constants of 8.592 × 10^4^ and 6.983 × 10^4^ at 293 K (20 °C) and 300 K (27 °C), respectively (Figure 8C), confirming that static quenching governs the interaction.

### 3.2. Förster Resonance Energy Transfer (FRET)

Förster resonance energy transfer (FRET) occurs when excited-state CDs act as energy donors and ground-state quenching agents serve as energy acceptors, provided two conditions are satisfied. FRET occurs when two key conditions are satisfied: (1) the emission spectrum of the energy donor overlaps with the absorption spectrum of the energy acceptor, and (2) the spatial separation between donor and acceptor is typically within 10–100 Å. This mechanism involves non-radiative energy transfer through long-range dipole–dipole coupling (Keesom-type interaction). During this process, the excited donor transfers energy directly to the acceptor without photon emission, leading to fluorescence quenching of the donor. Because FRET competes with radiative recombination, it generally results in a shortened fluorescence lifetime of CDs [86,87,88,89].

**Figure 9:**
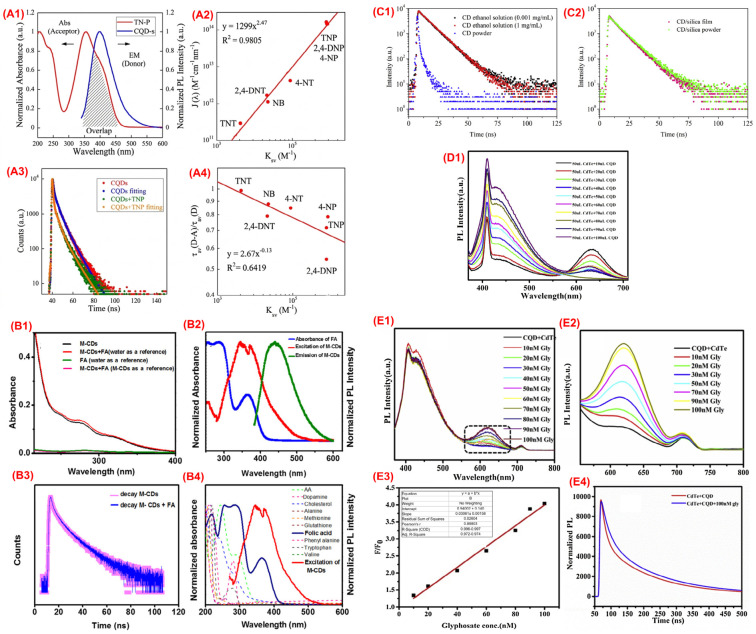
Correlation among fluorescence quenching behavior, spectral overlap, and fluorescence lifetime variation: (**A1**) Comparison between the absorption spectrum of TNP and the emission spectrum of CQDs; (**A2**) Relationship between the Stern–Volmer quenching constant (KSV) and the spectral overlap integral J(λ); (**A3**) Fluorescence lifetime decay curves of CQDs before and after the addition of TNP; (**A4**) Correlation between KSV and fluorescence lifetime variation expressed as τD−A/τD [87]. (**C1**) Fluorescence decay characteristics and corresponding fitting curves of carbon dots (CDs) in ethanol at concentrations of 0.001 and 1 mg mL^−1^, as well as CD powder; (**C2**) Fluorescence decay behavior and fitted curves of CD/silica film and CD/silica powder measured under 375 nm laser excitation [90]. (Color version available online.). (**B1**) UV–Vis absorption spectra of M-CDs using water as reference (black line), M-CDs mixed with folic acid (FA) using water as reference (red line), FA alone using water as reference (green line), and M-CDs + FA using M-CDs as reference (pink line); (**B2**) Spectral overlap among normalized FA absorption, excitation spectrum of M-CDs, and emission spectrum of M-CDs; (**B3**) Time-correlated single photon counting (TCSPC) fluorescence decay profiles of M-CDs (pink) and the M-CDs/FA mixture (blue); (**B4**) Overlap between absorption spectra of biomolecules and the excitation spectrum of M-CDs [93]. (**D1**) Fluorescence emission spectra of CdTe quantum dots (50 μL) recorded with increasing concentrations of CQD solution (**E1**,**E2**) Fluorescence response of CQD@CdTe composite solution; (**E3**) Corresponding linear detection range; (**E4**) Change in fluorescence lifetime of the CQD–CdTe sensing system after the introduction of glyphosate [94].

The FRET efficiency is highly dependent on the extent of spectral overlap between donor emission and acceptor absorption. Liang et al. [87] reported that the quenching efficiencies of nitroaromatic analytes toward chitosan-derived CQDs followed the sequence: 2,4,6-trinitrophenol (TNP) < 2,4-dinitrophenol (2,4-DNP) < p-nitrophenol (4-NP), which was inconsistent with a simple electron transfer process. Further spectral analysis showed strong overlap between the CQD emission spectrum and the absorption spectrum of TNP (Figure 9A1). Moreover, a strong linear relationship was observed between the spectral overlap integral J(λ) and the Stern–Volmer constant KSV (Figure 9A2). The presence of TNP also reduced the fluorescence lifetime of CQDs (Figure 9A3), and the ratio of fluorescence lifetimes (τD-A/τD) showed a good correlation with KSV (Figure 9A4), confirming that FRET is the dominant quenching pathway.

FRET is also closely associated with aggregation-induced quenching (ACQ) of CDs. Wang et al. [90] proposed two explanations for fluorescence quenching at high CD concentrations: (1) the proximity of CD particles falls within the Förster radius, enabling FRET and reducing fluorescence intensity [91]; and (2) self-absorption and re-emission of photons cause spectral overlap between absorption and emission (inner filter effect) [92]. Fluorescence lifetime measurements at different CD concentrations (Figure 9C) revealed significant differences, ruling out the inner filter effect and confirming that FRET plays the dominant role in ACQ.

### 3.3. Photoinduced Electron Transfer (PET)

The efficiency of PET is often governed by the energetic alignment between donor and acceptor species. Specifically, the feasibility of electron transfer depends on the relative positions of the highest occupied molecular orbital (HOMO) and lowest unoccupied molecular orbital (LUMO), as well as redox potentials. The driving force for PET can be estimated using Rehm–Weller-type considerations, where favorable free energy change enables efficient electron transfer. Therefore, electrochemical characterization (e.g., cyclic voltammetry) is often required to validate PET mechanisms [95,96]. Photoinduced electron transfer (PET) is essentially an electron transmission process between electron donors and acceptors upon light excitation. The formation of electron donor–acceptor complexes allow non-radiative ground-state recovery, resulting in fluorescence quenching [97]. In this mechanism, excited-state CDs act as electron donors (oxidative PET) or electron acceptors (reductive PET) [98]. PET leads to a decrease in CD fluorescence lifetime.

Bera et al. [94] reported that the fluorescence emission of CdTe quantum dots at 619 nm was quenched by chitosan-derived CQDs through PET (Figure 8C3). The addition of glyphosate (GP) to the CdTe-CQD mixed solution induced interactions between positively charged nitrogen atoms in GP and carboxylate groups arranged the surface of mercaptopropionic acid (MPA)-modified CdTe, leading to the separation of CdTe-CQD pairs and inhibition of PET. As a result, the emission peak at 632 nm was significantly enhanced (Figure 8). No similar phenomena were observed when glyphosate was replaced with alanine or glycine, indicating the selectivity of this PET-based sensing system for glyphosate detection.

### 3.4. Inner Filter Effect (IFE)

IFE can be divided into primary and secondary effects. Primary IFE occurs when the quencher absorbs excitation light, whereas secondary IFE involves absorption of emitted fluorescence. The extent of IFE depends on absorber concentration, optical path length, and spectral overlap. To avoid misinterpretation, fluorescence intensity should be corrected using absorbance-based correction equations. Additionally, front-face fluorescence geometry is recommended for highly absorbing systems to minimize reabsorption artifacts [99].

The inner filter effect (IFE) is caused by either the attenuation of excitation light or the absorption of emitted radiation by quenching agents. The former arises from the competitive absorption of excitation light by the quencher, reducing the actual light intensity acting on CDs. The latter involves the absorption of emitted fluorescence by the quencher, which not only decreases the fluorescence intensity but also induces non-trivial spectral changes. Unlike FRET (non-radiative energy transfer), IFE is a radiative process involving photon emission and reabsorption, so the fluorescence lifetime of CDs is largely unaffected by IFE [92].

Raveendran et al. [93] investigated the fluorescence quenching behavior of folic acid (FA) toward mint leaf-derived carbon polymer dots (M-CDs). The measured fluorescence lifetimes of M-CDs showed minimal variation after FA addition, changing from 5.19 ns to 5.41 ns (Figure 9B3). This negligible lifetime change excludes quenching pathways associated with FRET, electron transfer, and dynamic quenching. Furthermore, the UV–Vis absorption spectra of the combined M-CDs/FA system did not exhibit any additional absorption bands (Figure 9B1), indicating that ground-state complex formation, and therefore static quenching, was unlikely.

However, strong spectral overlap was observed between the absorption spectrum of FA and the excitation spectrum of M-CDs (Figure 9B2). This overlap suggests that the quenching process was primarily governed by the inner filter effect (IFE). In this case, FA absorbs a portion of the excitation light, which decreases the effective excitation intensity reaching the M-CDs and consequently reduces their fluorescence emission.

### 3.5. Experimental Discrimination of Quenching Mechanisms

In practical sensing systems, multiple quenching mechanisms may coexist, making it essential to distinguish them using experimental criteria. A combination of spectroscopic and physicochemical analyses is typically required. Fluorescence lifetime measurements provide a primary distinction: dynamic quenching, FRET, and PET usually shorten the excited-state lifetime, whereas static quenching and IFE generally do not affect lifetime. UV–Vis absorption spectroscopy helps identify ground-state complex formation, which is characteristic of static quenching. Stern–Volmer plots further aid interpretation; linear behavior often indicates a single mechanism, while deviation suggests combined static and dynamic quenching. Temperature and viscosity dependence also provide insight, as dynamic quenching is diffusion-controlled and increases with temperature, whereas static quenching decreases due to complex destabilization. For IFE, absorbance overlap between quencher and excitation/emission wavelengths must be evaluated, and correction methods based on absorbance should be applied to avoid misinterpretation (Table 2) [72].

## 4. Summary and Outlook

This review systematically summarizes the recent advances in the photoluminescence and quenching mechanisms of carbon dots (CDs). Carbon-core-state and surface-state emissions are the main luminescent mechanisms for carbon quantum dots (CQDs) and graphene quantum dots (GQDs), while molecular-state and crosslink-enhanced emissions are more prevalent in carbonized polymer dots (CPDs). Due to the significant influence of solvents on molecular-state luminescence, molecular-state emission and solvent effects are discussed together. Based on the action mode, crosslink-enhanced emission (CEE) is categorized into immobilization CEE and domain-limited CEE.

Fluorescence quenching of CDs can be achieved through five mechanisms: dynamic quenching, static quenching, Förster resonance energy transfer (FRET), photoinduced electron transfer (PET), and inner filter effect (IFE). Each mechanism has distinct characteristics in terms of fluorescence lifetime, absorption spectrum, and temperature dependence, which can be used for mechanism identification. A clear understanding of these quenching mechanisms provides a theoretical basis for the design and optimization of CD-based fluorescence sensors.

Despite significant progress in understanding CD photoluminescence and quenching mechanisms, several challenges remain:

(1) Elucidation of complex luminescent mechanisms: Most CDs exhibit multiple luminescent centers and mechanisms, and the synergistic effects between different mechanisms are not fully understood. Advanced characterization techniques (e.g., single-molecule spectroscopy, ultrafast transient absorption spectroscopy) and theoretical calculations (e.g., time-dependent density functional theory) should be combined to clarify the intrinsic luminescent mechanisms of CDs.
(2) Controllable synthesis of CDs: Based on the clear understanding of CD formation mechanisms, the balance between carbonization and polymerization should be precisely regulated to achieve controllable synthesis of CDs with specific structures and luminescent properties.
(3) Exploration of new luminescent phenomena: In addition to conventional fluorescence, CDs exhibit room-temperature phosphorescence (RTP) and thermally activated delayed fluorescence (TADF). Due to their long lifetimes, these luminescent phenomena have potential advantages in sensing, bioimaging, and optoelectronics. However, research on RTP and TADF mechanisms of CDs is still in its infancy, requiring further investigation.
(4) Development of high-performance sensors: Based on quenching mechanisms, rational design of CD structures and modification strategies should be carried out to improve the sensitivity, selectivity, and stability of sensors. The integration of CDs with other materials (e.g., metal–organic frameworks, hydrogels) may open new avenues for the development of multifunctional sensors, including environmental sensors, biosensors, and chemical sensors, based on fluorescence response mechanisms.

Carbon dots have been widely applied in fluorescence-based sensing due to their tunable emission, high stability, and surface functionalization capability. They have demonstrated excellent performance in detecting metal ions (e.g., Fe^3+^, Hg^2+^), small molecules (e.g., glucose, dopamine), and biomolecules (e.g., DNA, proteins). The sensing mechanisms typically involve fluorescence quenching processes such as dynamic quenching, static quenching, FRET, or PET, depending on the interaction between CDs and target analytes. These properties make CDs promising candidates for environmental monitoring, biomedical diagnostics, and food safety applications.

In conclusion, with the deepening of research on CD photoluminescence and quenching mechanisms, CDs are expected to find broader applications in sensing, bioimaging, optoelectronic devices, and other fields. Future research should focus on addressing the aforementioned challenges, promoting the practical application of CDs.

## Figures and Tables

**Table 1:** Photoluminescence Mechanisms of CDs.

Mechanism	Key Experimental Signature	Dependence	Origin	Typical Evidence
Carbon-core emission	Size-dependent emission	Weak solvent dependence	sp^2^ conjugated domains	TEM size vs. emission shift
Surface-state emission	Excitation-dependent emission	Surface chemistry	Defects, functional groups	XPS, FTIR, doping analysis
Molecular-state emission	Excitation-independent emission	Strong solvent dependence	Molecular fluorophores	Chromatography, NMR
Crosslink-enhanced emission (CEE)	Enhanced emission after rigidification	Aggregation/crosslinking	Polymer/sub-fluorophores	Temperature dependence, DFT

**Table 2:** Diagnostic Criteria for Quenching Mechanisms.

Mechanism	Lifetime Change	UV–Vis Absorption	Stern–Volmer Behavior	Temperature Effect	Key Identifier	Common Pitfall
Dynamic quenching	Decreases	No change	Linear	Increases	Diffusion-controlled	Confused with PET
Static quenching	No change	New absorption (complex)	Linear/nonlinear	Decreases	Ground-state complex	Overlap with IFE
FRET	Decreases	Spectral overlap required	Linear	Weak dependence	(distance)-dependent	Needs overlap confirmation
PET	Decreases	Sometimes	Nonlinear	Depends	Redox-driven	Overlaps with DQ
IFE	No change	Strong overlap	Apparent linear	No effect	Optical absorption	Misidentified as quenching

## Data Availability

No new data were created or analyzed in this study. Data sharing is not applicable to this article.
